# 

**Published:** 1848-01-01

**Authors:** 


### CONTENTS OF VOL. I.

### Analytical Reviews.

Page

Seymour on the Sedative Treatment of Insanity .................... 1
[illegible]r. Solly on the Physiology and Pathology of the Human Brain ...... 13
On the Condition of Lunacy in England .............................. 22
On Criminal Insanity ................. 38
Dr. Stroud on the Death of Christ ... 48
Dr. Webster on the Statistics and Pathology of Insanity ................. 57
Dr. Conolly on the Management of Lunatic Asylums .................. 71
Insanity in the State of New York ... 87
Insanity in Switzerland .............. 92
Baron Rolfe's Charge in the Case of the Boy Allnutt—The Plea of Insanity in Criminal Cases ......... 193
Dr. Wigan on the Duality of the Mind 218
Dr. Ideler on Religious Insanity ...... 229
Dr. Baron Ernst von Feuchtersleben on the Principles of Medical Psychology .............................. 247
M. Hubert Rodrigues on the General Paralysis of the Insane ............ 345
Dr. Moore on the Power of the Soul over the Body ..................... 366
[illegible]ate of Lunacy in the British Asylums 374
M. Belhomme on Political and Epidemic Insanity ................... 406
Mr. Taylor on Poisons in Relation to Medical Jurisprudence and Medicine .............................. 425
On the Education of Children predisposed to Insanity ............... 489
On German Psychology ............... 499
On the Physiology and Psychology of Apparitions, Dreaming, &c. ...... 512

Page

On Puerperal Insanity .................. 531
Is Insanity a Disease of the Blood ?... 541
Moral Physiology; or, the Priest and the Physician ........................ 557
Natural History of Man .............. 571
On the Transmission of Hereditary Qualities of Mind ................. 575
The Periodoscope ........................ 657

MISCELLANEOUS NOTICES ............ 579

### Original Communications.

Dr. Stubb's Visit to the Parisian Lunatic Asylums ........................ 100
Mr. Dendy on the Treatment of the Dying ................................ 112
Dr. Sigmond on the Treatment of Idiots ................................ 124
Dr. Reid on Puerperal Insanity ...... 128
On the State of Lunatic Asylums in Ireland ................................ 151
Dr. C. L. Robertson on the Treatment of Insanity resulting from Injury to the Head .......................... 155
On the Psychological Education of Medical Men ........................ 185
On Hereditary Insanity.................. 264
Notes on Feigned Insanity ............ 277
Puerperal Insanity........................ 284
On the Psychological Effects of certain Medicinal Agents ...................... 294
Malformation of the Brain ............ 306
Reports and Statistics of the German Hospitals for the Insane ............ 310
Case of Mania in a Child six years of age ...................................... 317

| | Page |
|---|---|
| Homicidal Insanity | 330 |
| Dr. Cattell on Insanity | 430 |
| Dr. Michéa on the Blood in the Neuroses | 436 |
| M. A. Brierre de Boismont on the Employment of Baths in Mania | 456 |
| The Influence of the Penitentiary System | 458 |
| Dr. Baillarger on the Pellagra | 460 |
| Correspondence from Paris | 466, 652 |
| Dr. Sigmond on Hallucinations | 585 |
| On Impulsive Insanity | 609 |
| Treatment of Idiots | 623 |
| Dr. Waller on Compression of the Carotids | 626 |

### Translations.

| | |
|---|---|
| On the Solitary System of Treating Prisoners | 159 |
| On the Influence of Music in Insanity | 166 |
| A Ball in a Lunatic Asylum | 168 |
| On the Exhibition of Ether in the Treatment of Insanity | 168 |
| Prize Question of the University of Berlin | 169 |
| Criminal Statistics of France | 657 |

### Medical Jurisprudence.

| | Page |
|---|---|
| Trial of Ovenston; with Remarks, by the Editor | 169 |
| Mr. Charles Pearson on the Treatment of Criminal Lunatics | 182 |
| Commission in Lunacy | 184 |
| Commission of Lunacy on Two Sisters | 318 |
| Commission of Lunacy on Miss Amelia Maria Hortensia Collings | 472 |
| Arson—Mental Alienation | 476 |
| Demande en Interdiction | 477 |
| Chelmsford—Charge of Murder—Acquittal on the ground of Puerperal Insanity | 478 |
| Maidstone—Acquittal of the Charge of Murder on the ground of Insanity | 480 |
| Remarks on the above Cases | 481 |
| Sir G. Stephen on the Plea of Monomania | 483 |
| A Will Contested on the Plea of Insanity of the Testator | 486 |
| Cour d'Appel at Paris | 634 |
| A Case of Monomania | 641 |

### Correspondence.

| | |
|---|---|
| Suicide—Life Assurance | 328 |

---

| | |
|---|---|
| Extracts and Varieties | 334 |
| Miscellaneous | 340, 469 |
| Medical Intelligence | 188 |
| To Correspondents | 192, 487 |

---

MONOGRAPH, No. I.—On the Cerebral Diseases of Children, particularly in reference to the Early Symptoms and Treatment. By WALTER C. DENDY, Esq.

